# The Administrative Side of Sanatorium Work

**Published:** 1909-08-14

**Authors:** 


### Nursing Administration.

### THE ADMINISTRATIVE SIDE OF SANATORIUM WORK.

### III.—WORK AS TREATMENT.

The great danger of the sanatorium undertaken by municipal effort is that cases may be despatched to the working colony for whom the system is useless, if not actually harmful. The particular form of treatment which relies upon active exercise combined with open air is still in its experimental stages, and there can be no doubt that it produces its best results in connection with a hospital for diseases of the chest, and when work can be regarded strictly as a therapeutic agency. The work carried on under Dr. Patterson's superintendence at Frimley first demonstrated the enormous healing force of man's natural activities, scientifically directed, in the cure of disease, and Frimley still leads the way in the science of applying natural means to the great end of health. Working as the Frimley colony does, as a branch of the Brompton Hospital for Consumption, it is possible there to extend the benefit of graduated labour to patients who would be completely out of place in a small sanatorium unprovided with a permanent fully trained staff, and aiming only at arresting the disease in its opening stages. This distinction must be borne well in mind by promoters of sanatorium extension. Cases may be easily quoted in which the labour treatment has proved eminently successful in cases of advanced consumption. But to argue from these successes that advanced phthisical patients can properly be received in the simple kind of sanatorium to which these articles relate, would be to expose the sufferers to grave danger and go far to discredit the whole system. The medical authority selected to examine and select patients for the sanatorium ought, if possible, to have experience in this form of treatment. He ought to have full powers to send only such patients as in his judgment are likely to be benefited by the graduated labour provided, and he must guard against the temptation of allowing the patient's distressing circumstances to weigh with him in his decision. There is more at stake in this matter than the individual patient. It goes without saying that the daily supervision of a competent medical man is an absolute *sine qua non* in the sanatorium, although in small establishments it is neither possible nor at all necessary to secure a resident.

When the right kind of patients have been secured (their name is legion), and when arrangements have been made for placing them under expert medical supervision, it will at once become apparent that they fall into several distinct categories. A certain proportion, including by far the greater number on first arrival, will be marked out for absolute rest until temperature and pulse indicate that the more regular treatment may safely be begun. After this stage is reached there are various grades through which the patient who responds satisfactorily to the treatment slowly progresses.

The following is the scheme of work as arranged at the Maitland Sanatorium:

Classes of Graduated Work.

(*a*). Cleaning of silver, brass, copper, basins, lamps, etc.; other light domestic work.

(*b*). Light gardening: Using hoe and rake, sweeping, lawn cutting, weeding, etc.; pony rolling; window and paint cleaning; painting or tarring; small repairs.

(*c*). Light spade work; heavy spade work; wheeling barrows; screening earth; path making; chopping wood; carpentering; farm work.

The scheme of graduated work is divided into time stages as follows:—For patients in Class A, $1\frac{1}{2}$ hours (9 to 10.30 a.m.); for patients in Classes B and C (1), $2\frac{1}{2}$ hours (9 to 11.30); (2) 4 hours (9 to 11.30 and 2 to 3.30); (3) 6 hours (9 to 11.30 and 2 to 3.30 and 4 to 5.30); for the working staff, 8 hours (7 a.m. to 5.30 p.m., less $2\frac{1}{2}$ hours for meals.

The following is a brief epitome of the system at Frimley:

The grades of work and exercise are: (1) Patients unfitted for active exercise make mops, mats, sew, etc.; (2) walking from one to six miles a day; (3) carrying baskets of mould, watering plants, weeding, etc.; (4) using a small shovel, etc.; (5) using a large shovel and mowing grass; (6) trenching, etc.; (7) as soon as a patient is considered fit to be discharged he is put to work at his trade, if he has one, for six hours a day for three weeks, in order to get the necessary muscles again used to the work.

As an example of what can be done by utilising labour intended primarily for the benefit of the individual, and only secondarily for profit, the record of the achievements of the Frimley patients is most instructive:

The Garden.—The soil is light and very poor, and requires a considerable amount of "working" before it becomes productive. The drain pipes among the flowers are to enable the plants to be watered at their roots, even though the sun is out. The whole of the grounds are kept in order by the patients. No money is spent on labour. The patients have completed a concrete reservoir, to hold half a million gallons of roof and surface water, for use in the baths and as a storage in case of fire. One thousand nine hundred and fifty-six working hours were occupied in its completion. The total weight of the concrete mixed and wheeled into position by the patients being 996 tons. The amount of earth excavated and carted away by the patients' own labour amounted to 4,175 tons.

The following work has been carried out by the patients since the opening of the Sanatorium: (1) Digging, manuring, and sowing over an acre of grass; (2) painting the outside of the woodwork of the buildings, and the whole of the inside of the administrative block, as well as some of the wards; (3) making most of the paths; (4) laying the concrete walk to the dining hall; (5) making a concrete subway, 150 yards in length, from the engine-room to the kitchen; (6) clearing a 20-foot "fire zone" round the boundary; (7) trenching and sifting about $3\frac{1}{4}$ acres of land; (8) getting and sifting gravel for the paths; (9) making the terrace and rock garden round the tennis court by the medical officers' house; (10) making the bank round the grounds; (11) felling and cutting into firewood about 200 trees; (12) laying out a considerable portion of the grounds; (13) building the greenhouse; (14) making the whole of the seats and poultry appliances; (15) laying down 1,500 feet of high-pressure fire mains with hydrants.

The secret of success in the labour department is organisation inspired by personal devotion. Routine without the gift of individual sympathy and the glow supplied by enthusiasm becomes cold, lifeless, and burdensome. Enthusiasm without the gifts of order and foresight is worse than officialism, it is positively dangerous. It is the happiness of sanatorium work to have enlisted both hearts and talents in its service.

